# A single institutional experience of thymic epithelial tumours over 11 years: clinical features and outcome and implications for future management

**DOI:** 10.1038/sj.bjc.6603833

**Published:** 2007-06-26

**Authors:** H-S Lee, S T Kim, J Lee, Y S Choi, J-H Han, Y-C Ahn, K-S Lee, J S Ahn, M J Ahn, K Kim, Y M Shim, J Kim, K Park

**Affiliations:** 1 Department of Thoracic Surgery, Samsung Medical Center, Sungkyunkwan University School of Medicine, Seoul, Korea; 2 Department of Hematology-Oncology, Samsung Medical Center, Sungkyunkwan University School of Medicine, Seoul, Korea; 3 Department of Pathology, Samsung Medical Center, Sungkyunkwan University School of Medicine, Seoul, Korea; 4 Department of Radiation Oncology, Samsung Medical Center, Sungkyunkwan University School of Medicine, Seoul, Korea; 5 Department of Radiology, Samsung Medical Center, Sungkyunkwan University School of Medicine, Seoul, Korea

**Keywords:** thymic epithelial tumour (TET), Masaoka's staging system, WHO histologic classification

## Abstract

Thymic epithelial tumours (TETs), the most common tumour of the anterior mediastinum, are epithelial neoplasms of the thymus with a wide spectrum of morphologic features. We retrospectively analysed clinical features of TET and the correlation of World Health Organisation (WHO) histologic classification and Masaoka staging system with different treatment modalities in 195 patients, from 1995 to 2005. According to the Masaoka's staging system, there were 78 (40.0 %) patients with stage I, 38 (19.5%) with stage II, 41 (21.0%) with stage III, 38 (19.5%) with stage IV. All patients were reclassified according to the WHO criteria as follows: Type A (*n*=9, 4.6%), AB (*n*=37, 18.9%), B1 (*n*=29, 14.8%), B2 (*n*=48, 24.6%), B3 (*n*=40, 20.5%), C (*n*=32, 16.4%). There was a fairly good correlation between Masaoka staging and WHO histotype (*P*<0.05). However, in multivariate analysis, the tumour stage and WHO histotype were two independent factors separately for predicting overall survival (*P*<0.001, *P*<0.001, respectively). Thus, both Masaoka stage and WHO histotype should be considered in risk stratification of therapy for TET patients. Patients with completely resected types B2, B3 and C and adjuvant radiotherapy (*n*=57) had more favourable disease-free and overall survival as compared with those without adjuvant treatment (*n*=20) (*P*=0.015, 0.015, respectively). Given that the predominant sites of recurrence after surgery was pleura/pericardium and lung, and the fact that complete resection was a significant influential factor for survival at log–rank test, an active investigation of newer treatment strategies such as neoadjuvant treatment to improve the resectability and development of optimal adjuvant treatment modality is a high priority especially for those with high-risk for recurrence or in patients with advanced stage disease.

Thymic epithelial tumours (TETs), the most common tumour of the anterior mediastinum, are epithelial neoplasms of the thymus with a wide spectrum of morphologic features (Morgenthaler *et al*, 1993; Loehrer *et al*, 2001). The incidence rates of TET are 0.5 in men and 0.3 in women in the United States per 100 000 populations (Sunpaweravong and Kelly, 2004). One-third to one-half of patients present with asymptomatic anterior mediastinal mass on chest radiography, one-third present with local symptoms (e.g. cough, chest pain, superior vena cava syndrome, and dysphagia) and one-third are detected during the evaluation of myasthenia gravis (MG) (Thomas *et al*, 1999). Approximately 10–15% of patients with MG will have TET and MG occurs in about 30% of patients with TET (Kornfeld *et al*, 1978; Pescarmona *et al*, 1990; Wilkins *et al*, 1991; Blumberg *et al*, 1995; Thomas *et al*, 1999; Sperling *et al*, 2003).

Owing to the rarity and its morphologic heterogeneity, it is only recent that provisional consensus on staging and histologic classifications have been reached. The most widely adopted staging system is the one proposed by Masaoka in 1981, which has demonstrated good prognostic relevance. Several groups, however, have reported several drawbacks of the Masaoka's staging system. First, the system does not effectively differentiate between stages I and II with prognostic significance. Secondly, the system is not well-suited for the staging of thymic carcinomas, which are now categorised as Type C in the World Health Organisation (WHO) classification of TET. The WHO proposed a histologic classification system, which subdivided TETs into three major categories, A, B, and C (thymic carcinoma) in 1999. Since the introduction of the WHO classification system, several studies have validated the predictability and good correlation with survival of TETs. For the WHO classification, some groups have reported conflicting results according to histotypes: few groups (Okumura *et al*, 2002; Nakagawa *et al*, 2003) concluded that type B3 thymoma conferred poorer survival compared to type B2, whereas the other reports (Chalabreysse *et al*, 2002; Chen *et al*, 2002; Rieker *et al*, 2002) showed no significant difference in survival between type B2 and B3 thymomas. Thus, the controversies in classifications of TETs are unsettled at this time.

We retrospectively analysed the clinical features of TETs and investigated the prognostic impact of the WHO histotype, Masaoka's staging system and the complete resectability in a series of 195 patients with TETs over a period of 11 years at a single institution.

## PATIENTS AND METHODS

### Patients

From 1995 to 2005, 195 patients were pathologically confirmed of TET at the Samsung Medical Centre. Histologic types of TET were reviewed by one pathologist specialised in thymic tumours and re-classified according to WHO criteria as: type A (medullary, spindle cell), type AB (mixed), type B (cortical) with subtypes B1, B2 and B3, and type C. The following clinical data were collected from medical records for each patient: physical examination, surgical and pathologic reports, imaging and treatment modalities. The stages were classified according to the Masaoka's staging system, which was originally described by surgical stage, that is, stage I, microscopically encapsulated tumour; stage II, microscopic invasion into capsule; stage III, macroscopic invasion into neighbouring organs; stage IVa, pleural or pericardial dissemination; stage IVb, lymphatic or haematogenous metastasis (Masaoka *et al*, 1981).

### Treatment

Total thymectomy was defined as the resection of the entire thymus and mediastinal fatty tissue between both phrenic nerves; thymomectomy was defined as the resection of thymoma leaving residual thymic tissue behind; complete resection was defined as no macroscopic or microscopic residual tumour; and incomplete resection was defined as the documented macroscopic or microscopic residual tumour. The postoperative radiation therapy at the anterior mediastinum was routinely given to the patients with WHO classification B2 or greater. Before 1999, the indication for adjuvant radiation therapy was Masaoka stage II or higher, as well as non-medullary type of Muller–Hermelink classification. After the surgery, patients were monitored every 3–4 months with chest CT scan for 2 years, every 6 months for 3 years, and yearly thereafter.

The cyclophosphamide (PAC) chemotherapy regimen consisted of PAC 500 mg m^−2^ (over 1 h), doxorubicin 50 mg m^−2^ (over 30 min), and cisplatin 50 mg m^−2^ (over 1 h) administered on day 1 and repeated every 3 weeks. All patients received adequate hydration (pre- and post-cisplatin) with at least 3 l normal saline in 24 h. The recommended anti-emetic schedule comprised 5-HT3 inhibitors and dexamethasone 20 mg at the beginning of cisplatin infusion. In the adjuvant setting, a total of three cycles were administered post-operatively. In cases of post-operative combined chemotherapy and radiotherapy, radiotherapy was administered first and then followed by three cycles of PAC. For palliative chemotherapy, PAC chemotherapy was given until the documented progressive disease or up to six cycles.

Radiotherapy was administered with a 6∼10 MV linear accelerator. Adjuvant radiation therapy was given 4–6 weeks after surgery at a total dose of 54 Gy (1.8–2 Gy daily, five times/week). In palliative setting, primary radiation therapy was administered at a daily dose of 2.5–3 Gy for a target dose of greater than 30 Gy. Under the conditions where performance permits and extrathoracic disease is controlled, primary radiation therapy up to 50–60 Gy was given. Selected patients with initially unresectable disease received two to four cycles of PAC chemotherapy and for patients with stable, partial or complete response to chemotherapy, patients further received a total radiation dosage of 50–60 Gy to the primary tumour and regional lymph nodes.

### Pathology

In each case, one pathologist (J Han) evaluated the haematoxylin-eosin stained formalin-fixed paraffin sections of surgically resected thymoma specimens at the time of surgery and blindly reviewed all the pathologic specimens at the time of this study. All cases diagnosed before 1999 were initially categorised according to the Muller–Hermelink classification and reclassified using the WHO classification for the study. Immunohistochemical staining was used to distinguish TETs from other mediastinal tumours, using 1 : 50 CD5 (DAKO, Carpinteria, CA, USA), 1 : 100 cytokeratin (DAKO), 1 : 50 MIC-2 (CD99) (DAKO), 1 : 100 leucocyte common antigen (LCA) (DAKO) and 1 : 40 placental leucocyte alkaline phosphatase (PLAP) (Novocastra, Newcastle upon Tyne UK).

### Statistical analyses

The statistical difference of the average value was examined with the Student's *t*-test. Survival was calculated from the time of diagnosis to the last follow-up date or death, and survival curves were obtained by the Kaplan–Meier method; differences between two curves were assessed using the log–rank test. All the deaths that were not related to the tumour were considered as censored observations. All *P*-values are two-tailed. Prognostic factors were identified by multivariate analysis, using the Cox proportional hazards regression model. The following variables were considered as possible candidate prognostic factors: age, sex, presence of MG, completeness of the resection, Masaoka stage, and WHO histologic type. *P*<0.05 was assumed significant unless otherwise stated.

## RESULTS

### Patients characteristics

The characteristics of the 195 patients are shown in Table 1. Median age was 49 years (range, 18–81). The male/female ratio was 1.1 : 1.0. Sixty-one patients (31.3%) had invasion or metastases to other organs with lung (*n*=37, 19.0%) being the most common site. According to the Masaoka's staging system, there were 78 (40.0 %) patients with stage I, 38 (19.5%) with stage II, 41 (21.0%) with stage III, and 38 (19.5%) with stage IV. All patients were reclassified according to the WHO criteria as follows: Type A (*n*=9, 4.6%), AB (*n*=37, 18.9%), B1 (*n*=29, 14.8%), B2 (*n*=48, 24.6%), B3 (*n*=40, 20.5%), C (*n*=32, 16.4%) (Table 1). Twenty-seven patients (13.8%) presented with paraneoplastic syndromes such as MG, Cushing's syndrome (*n*=2, 1.0%), and haemolytic anaemia (*n*=2, 1.0%). MG was particularly frequent in type B2 TETs with statistical significance as compared to other histotypes (*P*=0.010).

### Association of WHO histologic subtypes with Masaoka stages

The association between the WHO histologic subtype and Masaoka stage was evaluated (Table 2). Two-thirds of the type A, AB, B1, and B2 TETs were categorised as Masaoka stages I and II. By contrast, types B3 and C were detected in advanced stages (stages III and IV) at high frequencies (68.1% at presentation; *P*<0.01). There was a significant correlation between Masaoka staging and WHO histotype (*P*<0.05) and the proportions of advanced stage tumours gradually increased from type A to type C. Nevertheless, there were discordances between Masaoka stages and WHO histologic subtypes in individual patients (Table 2). Some patients had advanced Masaoka stage for their benign histotype: five patients (17.2 %) with B1 tumour type had stage IV disease. In contrast, others had localised diseases despite of aggressive histologic subtype: seven patients (21.9%) of type C had stage I or II diseases, all of whom were treated with R0 resection±adjuvant therapy.

### Association of primary tumour resection with Masaoka stages and WHO histologic subtypes

One hundred and sixty-one (82.6%). of 195 patients received complete resections. Complete tumour resection (R0) was achieved in all the patients with stage I and II TETs and the rate of complete resection drastically decreased as stage increased (stage I, 100%; stage II, 100%; stage III, 85.3%; stage IV, 26.3%; Table 3). In all, 91 (56.5%) of the 161 patients with R0 resection received post-operative therapy such as chemotherapy, radiotherapy or chemoradiotherapy. The general indications for post-operative radiotherapy were as follows: positive resection margin, WHO subtype of B2 or greater, and/or Masaoka stage II or greater. Of the 91 patients who received adjuvant therapy following complete resections, 78 (85.7%) patients received post-operative radiotherapy alone. Nine patients with stage III/IV disease received post-operative systemic chemotherapy or chemoradiotherapy due to incomplete resections.

According to the WHO histotype, R0 resection was achieved in all type A and AB TETs and in most of type B1 TETs (93.1%). Of the 73 patients with type A, AB and B1 who underwent R0 resection, 21 (28.8%) patients received post-operative radiotherapy. By contrast, complete R0 in type B2, B3, and C TETs was achieved in only 81.2, 75.0 and 59.3% of cases, respectively (Table 4). Most of the patients who underwent R1 or R2 resections received post-operative therapy (chemotherapy or chemoradiotherapy). The frequency of the invasive tumours and the rate of incomplete resection were higher in type B3 and C than other histologic subtypes (*P*<0.01).

### Association of Masaoka stage and WHO histologic subtype with survival

**Figure 1:**
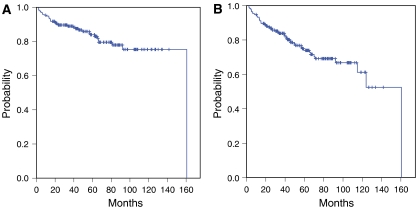
Overall survival (**A**) and disease-free survival (**B**) in thymic epithelial tumours.

**Figure 2:**
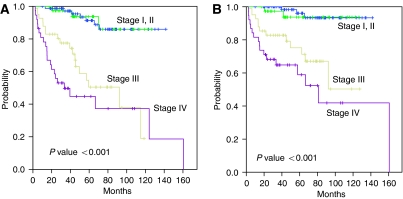
Disease-free survival (**A**) and overall survival (**B**) according to the Masaoka stage.

Tumour stage and WHO histotype were the most important factors predicting survival in TET patients. The 5-year survival rates were 96, 100, 71 and 52% for stages I, II, III and IV, respectively (Figures 1 and 2). There was no statistically significant difference in survival rates between Masaoka stage I and II tumours (*P*=0.063) and between stage III and IV tumours (*P*=0.13) (Figure 2). The 5-year survival rates were 100, 89, 89, 68, and 47% for the types A+AB, B1, B2, B3, and C, respectively. In multivariate analysis, the tumour stage and WHO histotype were two independent factors for predicting overall survival (*P*<0.001, *P*<0.001, respectively).

### Treatment modality and recurrence after complete resection

**Figure 3:**
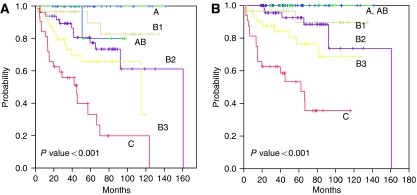
Disease-free survival (**A**) and survival (**B**) according to the WHO histologic classification.

**Figure 4:**
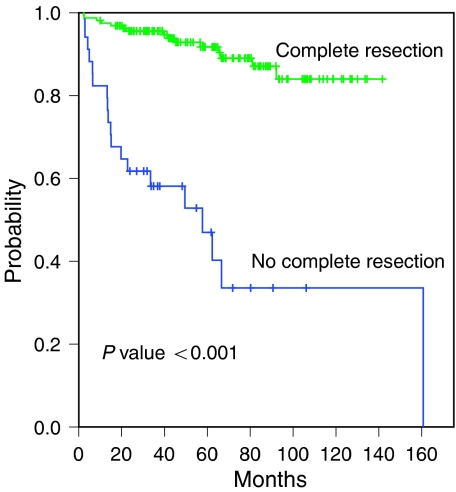
Survival according to the complete resection of thymoma.

Fifty (66.7%) of 75 patients with WHO type A, AB and B1 thymomas achieved long-term complete remissions with primary complete resection alone. The administration of adjuvant radiotherapy in patients with WHO type A, AB, and B1 did not significantly influence overall survival (*P*=0.185) or disease-free survival (*P*=0.962) when compared to those who did not receive adjuvant therapy during the same period of time. On contrary, in the WHO type B2 to C subgroup, adjuvant radiotherapy significantly improved overall survival (*P*=0.015) and disease-free survival (*P*=0.015) (Figures 3 and 4).

In 21 stage IV patients treated with primary chemotherapy or chemoradiotherapy without surgery, the 5-year survival rate was only 43% with median survival of 57.7 months (95% CI, 3.6–111.8 months). The regimen of chemotherapy used in all these patients was cisplatin, doxorubicin and PAC. In general, the patients with complete resection (*n*=161) had a significantly better survival than those without complete resection (*n*=34) (*P*<0.001) (Figure 3). Of the 161 patients with initial R0 resection, 19 patients have relapsed after a median follow-up duration of 77.6 months (45.3–137.3 months). The first recurrent sites after R0 resection were as follows in order of frequency: pleura (*n*=10), lung (*n*=4), local (*n*=3), bone (*n*=1) and liver (*n*=1).

## DISCUSSION

In the present study, the histologic subtypes based on the WHO classification were determined in all of the 195 patients. The most frequent histologic subtype was type B2 (24.6%), followed by type B3 (20.5%). The incidence of type AB was slightly lower (37 cases, 18.9%) compared to other studies (Fang *et al*, 2005; Kim *et al*, 2005), which reported the proportion of type AB to be 23 to 32%. MG was encountered in 27 (13.8%) of the 195 TET patients (two patients of type AB, seven of B1, 10 of B2, and eight of type B3) MG was significantly more frequent in type B than in types A and AB (*P*<0.01). A similar distribution of MG was reported in other series (Chalabreysse *et al*, 2002; Chen *et al*, 2002; Okumura *et al*, 2002; Nakagawa *et al*, 2003). Although MG was particularly frequent in type B2 TETs, there was no difference in survival between those with and without MG; *P*>0.05). Distant metastases were distinctly uncommon at initial presentation with this tumour and the most commonly involved site was pleura followed by pericardium, lung, bone and liver.

We investigated the correlation between the WHO histologic subtypes and the Masaoka's staging system. Thirty (24.4%) of 123 patients with types A, AB, B1 and B2 had advanced disease (stages III and IV), while 49 (68.1%) of 72 patients with types B3 and C had advanced disease (Table 2). It has been also reported that most of type A, AB, B1 and B2 TETs behave in a benign fashion but type B3 and C have to be considered malignant tumours with a potential to metastasize (Truong *et al*, 1990; Hsu *et al*, 1994). The long-term outcome of TET patients was significantly correlated to tumour stage, completeness of surgical resection, and WHO histotype (*P*<0.001). The overall survivals of stage III and IV tumours were significantly poorer than those of stage I and II tumours (*P*<0.05). The Masaoka’s staging system, however, did not significantly differentiate the survival outcome between the stage I and II patients in accordance with the previous studies (Pescarmona *et al*, 1990; Lardinois *et al*, 2000; Okumura *et al*, 2002; Nakagawa *et al*, 2003). Moreover, despite of clear association between Masaoka stages and WHO histologic subtypes, good proportions of patients showed discordances between the two systems: 17.2% of B1 tumour types with stage IV disease and 21.9% of type C with stage I/II disease. Since both Masaoka stage and WHO subtypes are independent prognostic factors for survival in TETs, these two parameters should be carefully incorporated in decision-making. Clearly, there is no established guideline for the discordant cases between the stage and the histologic subtypes. To enhance treatment outcome, risk stratification therapy of this subset of TET patients should be sought in future trials.

In the WHO histologic classification, type B3 thymoma is still a peculiar group that remains controversial in the literature. Several reports (Okumura *et al*, 2002; Nakagawa *et al*, 2003) have revealed poorer prognosis of type B3 thymoma compared to type B2, whereas the other reports (Chalabreysse *et al*, 2002; Chen *et al*, 2002; Rieker *et al*, 2002) showed no significant difference in survival between type B2 and B3 thymomas. Interestingly, when WHO subtypes were simplified into three groups (A-B2 *vs* B3 *vs* C), the survival curve showed more distinct pattern with type B3 being an intermediate prognostic group (Figure 3). These simplified three groups might be useful in the future research and/or therapeutic planning for TETs as reported by other studies (Rieker *et al*, 2002; Kim *et al*, 2005).

**Figure 5:**
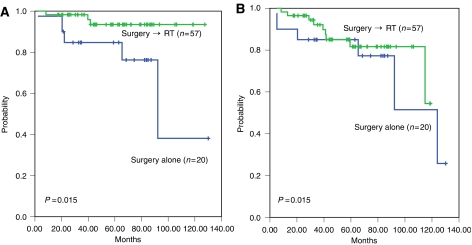
Influence of adjuvant radiotherapy on (**A**) disease-free survival (**B**) overall survival of patients with type B2-C TETs.

Post-operative radiotherapy has long been considered unnecessary for stage I TETs after complete resection, although there is controversy over adjuvant radiotherapy for stage II TETs (Maggi *et al*, 1991; Regnard *et al*, 1996). In view of our data, the adjuvant radiotherapy did not significantly influence on survival of patients with type A – B1 when compared to the patients with no adjuvant radiotherapy. Importantly, patients with completely resected types B2, B3 and C and adjuvant radiotherapy had more favourable disease-free and overall survival as compared with those without adjuvant radiotherapy (Figure 5). Nakahara *et al* (1988) reported 29% recurrence rate for patients with stage II thymoma after surgery alone as compared to 8% for those with surgery followed by adjuvant radiation therapy. Similarly, another study showed a recurrence rate of 28 *vs* 8% in stage II patients with post-operative radiation therapy and those without, respectively (Quintanilla-Martinez *et al*, 1994). Most recently, an Italian study demonstrated no difference in disease-free survival between 32 stage II patients with surgery alone and 26 patients with surgery followed by mediastinal radiation therapy (Rena *et al*, 2007). However, all of these studies including ours are retrospectively conducted, and thus, prospective randomised controlled studies are essential to define the definite role of adjuvant radiotherapy for patients with stage II or III TETs.

Only 10 out of 38 patients with stage IV TETs achieved R0 resection in our series and most stage IV patients received chemotherapy alone or combined chemotherapy and radiotherapy. For the patients who received chemotherapy, all received cisplatin, doxorubicin, and PAC regimen as the first-line therapy. In a prospective phase II trial by US co-operative groups (Loehrer *et al*, 1994; Oshita *et al*, 1995), the combination of cisplatin, doxorubicin, and PAC showed an overall response rate of 50% with median survival of 38 months. In our study, 5-year survival rates were 52% and the median overall survival was 66.1 months (95% CI, 36.6–95.6 months) in 38 patients with stage IV. For the 21 stage IV patients who were treated with primary chemotherapy or chemoradiotherapy without surgical resection, the 5-year OS was only 43% with median overall survival of 57.7 months (95% CI, 3.6–111.8 months), suggesting that the debulking operation might improve the survival of stage IV disease (*P*<0.05). However, Myojin and colleagues reported increased pleural dissemination and recurrence after surgical exploration for stage III TETs, and questioned the role of debulking surgery in this setting (Fang *et al*, 2005). Therefore, the role of debulking surgery for stage III/IV still remains to be defined.

Patients with incomplete resection had significantly worse overall survival than patients with a complete resection (*P*<0.05) and the WHO subtype was also an important prognostic factor in patients with incomplete resection. Most patients who underwent incomplete resection, WHO subtype B3 or C, and stage III or IV, died of progressive disease, despite multimodality treatment. After a median follow-up duration of 77.6 months (45.3–137.3 months), 19 of 161 patients have relapsed following R0 resection. Given that the predominant sites of recurrence after surgery were pleura/pericardium and lung, and the fact that complete resection was a significant prognostic factor for survival, focus on novel treatment strategies such as neoadjuvant treatment to improve the resectability and development of optimal adjuvant treatment modality is a high priority, especially in patients with high risk of recurrence or those with advanced stage disease. At present, a precise treatment algorithm for patients who could potentially benefit from preoperative chemotherapy and/or radiotherapy is not firmly established. Given the fact that most of our patients were selected for preoperative treatment based on CT findings, the superiority of PET-CT over CT in identifying potential candidates for preoperative treatment needs to be investigated.

Recently, Shin *et al* (1998) reported that 82% of 12 unresectable TETs were completely resected after induction chemotherapy, and another study (Venuta *et al*, 2003) reported an increased resection rate and prolonged survival for stage III and IV TETs following neoadjuvant chemotherapy with cisplatin, epirubicin, and etoposide compared to the historical control. In multivariate analysis, the tumour stage and WHO histotype were two independent factors for predicting overall survival (*P*<0.001, *P*<0.001, respectively). Thus, both Masaoka stage and WHO histotype should be considered in-risk stratification of therapy for TET patients.

In conclusion, although there was a clear association between the two systems, the Masaoka stage and WHO histotype were two independent prognostic factors for survival. Despite some discordance between the two systems, the two parameters, therefore, should be considered during decision-making for therapeutic approaches. Furthermore, patients with WHO subtypes B3 or C or stage III/IV pursued an aggressive clinical course despite of multimodality treatment implicating more innovative therapeutic approach such as neoadjuvant therapy with novel agents warrants further investigations. Operable patients should be stratified by risk for recurrence and be given adjuvant treatment with more tailored therapy.

## Figures and Tables

**Table 1:** Clinical findings of patients with TET

Median age (range)	49 years (18–81 years)
Gender ratio (M : F)	103 : 92 (1.1 : 1)
Myasthenia Gravis	27 (13.8%)
	
*Involved organ*	61 (31.3%)
Lung	37 (19.0%)
Pericardium	27 (13.8%)
Pleural seeding	20 (10.3%)
Innominate vein	16 (8.2%)
Superior vena cava	8 (4.1%)
Pericardial seeding	5 (2.6%)
Mediastinal lymph node	4 (2.0%)
Pulmonary vessels	3 (1.5%)
Phrenic nerve	2 (1.0%)
Chest wall	1 (0.5%)
Aorta	1 (0.5%)
Lung metastasis	1 (0.5%)
Bone metastasis	1 (0.5%)
Liver	1 (0.5%)
	
*Masaoka’s stage*	
I	78 (40.0%)
II	38 (19.5%)
III	41 (21.0%)
IV	38 (19.5%)
	
*WHO type*	
A	9 (4.6%)
AB	37 (18.9%)
B1	29 (14.8%)
B2	48 (24.6%)
B3	40 (20.5%)
C	32 (16.4%)

Abbreviation: TET=thymic epithelial tumours.

**Table 2:** Masaoka stage with reference to the WHO histologic classification system for thymic epithelial tumors

	**WHO tumour types**						
**Stage**	**A**	**AB**	**B1**	**B2**	**B3**	**C**	**Total**
I	5 (6.4)	28 (35.9)	12 (15.4)	20 (25.6)	10 (12.8)	3 (3.8)	78
II	2 (5.3)	9 (2.4)	10 (26.3)	7 (18.4)	6 (15.8)	4 (10.5)	38
III	2 (4.9)	0 (0.0)	2 (4.9)	9 (22.0)	12 (29.3)	16 (39.0)	41
IV	0 (0.0)	0 (0.0)	5 (13.2)	12 (31.6)	12 (31.6)	9 (23.7)	38
Total	9 (4.6)	37 (19.0)	29 (14.9)	48 (24.6)	40 (20.5)	32 (16.4)	195

Abbreviation: WHO=World Health Organisation.

**Table 3:** Distribution of treatment modalities according to the masaoka tumour stage

	**Masaoka stage**				
	**I**	**II**	**III**	**IV**	**Total**
*n* (%)	78 (40.0)	38 (19.5)	41 (21.0)	38 (19.5)	195
					
*Resection type*, n (%)					
Complete	78 (100.0)	38 (100.0)	35 (85.3)	10 (26.3)	161 (82.5)
Incomplete	0 (0.0)	0 (0.0)	4 (36.4)	7 (63.6)	11 (5.6)
Biopsy only	0 (0.0)	0 (0.0)	2 (8.7)	21 (91.3)	23 (11.8)
					
*Treatment modalities*, n (%)					
R0 only	43 (55.1)	19 (50.0)	5 (12.2)	3 (7.9)	70 (35.9)
R0+RT	35 (44.9)	17 (44.7)	23 (56.1)	3 (7.9)	78 (40.0)
R0+CT	—	2 (5.3)	—	3 (7.9)	5 (2.6)
R0+CRT	—	—	7 (17.1)	1 (2.6)	8 (4.1)
R1/R2+RT	—	—	2 (8.7)	1 (2.6)	3 (1.5)
R1/R2+CT or CRT	—	—	2 (8.7)	4 (10.5)	6 (3.1)
CT only	—	—	1 (2.4)	16 (42.1)	17 (8.7)
CRT only	—	—	1 (2.4)	5 (13.1)	6 (3.1)
R1/R2 alone	—	—	—	2 (5.3)	2 (1.0)

Abbreviations: CT=chemotherapy; CRT=chemoradiation; R0=R0 resection; RT=radiation therapy; R1=R1 resection; R2=R2 resection.

**Table 4:** Distribution of treatment modalities according to the WHO subtypes

	**WHO tumour types**						
	**A**	**AB**	**B1**	**B2**	**B3**	**C**	**Total**
n (%)	9 (4.6)	37 (18.9)	29 (14.8)	48 (24.6)	40 (20.5)	32 (16.4)	195 (%)
							
*Resection type*, n (%)							
Complete	9 (100.0)	37 (100.0)	27 (93.1)	39 (81.2)	30 (75.0)	19 (59.3)	161 (82.5)
Incomplete	—	—	1 (3.4)	4 (8.3)	3 (7.5)	3 (9.4)	11 (5.6)
Biopsy only	—	—	1 (3.4)	5 (10.4)	7 (17.5)	10 (31.3)	23 (11.8)
							
*Treatment modalities*, n (%)							
R0 only	6 (66.7)	31 (83.8)	13 (44.8)	14 (29.2)	4 (10.0)	2 (6.3)	70 (35.9)
R0+RT	3 (33.3)	6 (16.2)	12 (41.4)	24 (50.0)	22 (55.0)	11 (34.4)	78 (40.0)
R0+CT	—	—	2 (6.9)	1 (2.1)	1 (2.5)	1 (3.1)	5 (2.6)
R0+CRT	—	—	—	—	3 (7.5)	5 (15.6)	8 (4.1)
R1/R2+RT	—	—	—	1 (2.1)	—	2 (6.3)	3 (1.5)
R1/R2+CT or CRT	—	—	—	2 (4.2)	3 (7.5)	1 (3.1)	6 (3.1)
CT only	—	—	1 (3.4)	4 (8.3)	6 (15.0)	6 (18.8)	17 (8.7)
CRT only	—	—	—	1 (2.1)	1 (2.5)	4 (12.5)	6 (3.1)
R1/R2 alone	—	—	1 (3.4)	1 (2.1)	—	—	2 (1.0)

Abbreviations: CT=chemotherapy; CRT=chemoradiation; R0=R0 resection; RT=radiation therapy; R1=R1 resection; R2=R2 resection.
